# MiR-106a aggravates sepsis-induced acute kidney injury by targeting THBS2 in mice model^1^

**DOI:** 10.1590/s0102-865020190060000002

**Published:** 2019-08-19

**Authors:** Yezhou Shen, Jiaoyang Yu, Yunyan Jing, Jian Zhang

**Affiliations:** I Bachelor, Intensive Care Unit, The Affiliated Hospital of Hangzhou Normal University, Hangzhou, China. Conception and design of the study, acquisition of data, technical procedures, manuscript preparation and writing.; II Master, Intensive Care Unit, The Affiliated Hospital of Hangzhou Normal University, Hangzhou, China. Technical procedures, acquisition of data.; III Bachelor, Intensive Care Unit, The Affiliated Hospital of Hangzhou Normal University, Hangzhou, China. Statistical analysis, interpretation of data.

**Keywords:** Sepsis, Acute Kidney Injury, Lipopolysaccharides, Mice

## Abstract

**Purpose:**

To investigate the role and related mechanisms of miR-106a in sepsis-induced AKI.

**Methods:**

Serum from sepsis and healthy patients was collected, sepsis mouse model was established by cecal ligation and puncture (CLP). TCMK-1 cells were treated with lipopolysaccharide (LPS) and transfected with THBS2-small interfering RNA (siTHBS2), miR-106a inhibitor, miR-106a mimics and their negative controls (NCs). The expression of miR-106a, thrombospondin 2 (THBS2), Bax, cleaved caspase-3 and Bcl-2, cell viability, relative caspase-3 activity and TNF-α, IL-1β, IL-6 content were respectively detected by quantitative real-time polymerase chain reaction (qRT-PCR), western blotting, Cell Counting Kit-8 (CCK-8) and enzyme linked immunosorbent assay (ELISA). The relationship between miR-106a and THBS2 was confirmed by dual luciferase reporter assay.

**Results:**

MiR-106a was up-regulated in serum of sepsis patients, CLP-induced mice models and LPS-induced TCMK-1 cells. LPS reduced cell viability and Bcl-2 expression, and increased caspase-3 activity, Bax expression, the content of TNF-α, IL-1β, IL-6. THBS2 was a target of miR-106a. The decreases of caspase-3 activity, TNF-α, IL-1β, IL-6, Bax expression and the increases of cell viability, Bcl-2 expression caused by miR-106a knockdown were reversed when THBS2 silencing in LPS-stimulated TCMK-1 cells.

**Conclusion:**

MiR-106a aggravated LPS-induced inflammation and apoptosis of TCMK-1 cells via regulating THBS2 expression.

## Introduction

Sepsis is a systemic inflammatory response syndrome caused by infection or highly suspicious infection, and is also a major cause of multiple organ failure and septic shock until death^1,2^. The uncontrolled inflammatory response and immune dysfunction of the body are the main pathophysiological basis of sepsis^2^. The degree of excessive inflammation and immune dysfunction directly affect the occurrence and development of sepsis^2^. Moreover, acute kidney injury (AKI) is one of the most common organ failure symptoms in sepsis^3^. The pathways of AKI induced by sepsis may include coagulopathy, inflammation, oxidative stress and changes in renal tubular epithelial cells to injury^4^. However, the mechanism of these effects is complex and there is no clear research report at present.

MicroRNAs (miRNAs) are non-coding endogenous RNAs that exert translational inhibition by targeting messenger RNA (mRNA), and have been shown to regulate various stages of sepsis^5-7^. A number of studies have shown that miRNAs disorders are associated with clinical symptoms of inflammation and sepsis^6-8^. For example, the up-regulation of miR-21-3p promotes sepsis-induced cardiac dysfunction^6^. MiR-15a/16 is up-regulated in serum of neonatal sepsis patients, and inhibits LPS-induced inflammatory signaling pathway activity^7^. MiR-128 expression decreased, while miR-21 levels increased in serum and LPS-induced podocytes of patients with sepsis, leading to podocyte damage in sepsis^8^. The above studies show that miRNAs play a crucial role in regulating immune response and sepsis. Therefore, understanding the potential mechanism of miRNAs in sepsis will help to better treat this disease. In addition, it has been reported that miR-106a expression is up-regulated in the serum of sepsis mice and inflammatory bowel disease mice model, and then the absence of miR-106a can alleviate the inflammatory response^9,10^. However, the role of miRNA-106 in sepsis-induced AKI remains unclear.

Thrombospondins (THBSs) is a protein family with a minimum of five members that participate in a variety of biological processes by combining some target proteins, such as cell motility, apoptosis, cytoskeletal formation, and can also serve as a response platform for extracellular matrices^11,12^. THBS2 is a relatively special member of THBSs family that has anti-angiogenic effects and interacts with various cellular receptors and growth factors to regulate cell proliferation, apoptosis and adhesion^13^. THBS2 has also been reported to act as important downstream targeting gene of miRNAs to form a miRNAs/THBS2 network for various diseases, such as cervical cancer, human myxoid liposarcoma and renal cancer^14-16^.

In this study, we collected serum from sepsis and healthy patients, constructed sepsis mouse models by cecal ligation and puncture (CLP) and stimulated mouse kidney epithelial TCMK-1 cells with LPS *in vitro* to investigate the role of miR-106a in sepsis-induced AKI and its related mechanisms, as well as the possible regulatory relationship with THBS2.

## Methods

Between March, 2018 and January, 2019, a total of 50 patients (34 males and 16 females, mean age: 48±7.21 years) with sepsis who lived in Intensive Care Unit (ICU) and 30 healthy controls (21 males and 9 females, mean age: 42±5.46 years) were recruited in this study from The Affiliated Hospital of Hangzhou Normal University. The diagnosis of sepsis complied with the American College of Chest Physicians (ACCP) and the Society of Critical Care Medicine (SCCM) joint definition of sepsis diagnostic criteria in US^17^.

The venous blood (5 mL) was collected from patients with sepsis and healthy people, and then the whole blood was preserved by anticoagulation with 1 mL of Edathamil (EDTA-K2, Solarbio, Beijing, China). Serum was obtained by centrifugation after 4 mL EDTA-K2 coagulation and stored at -20°C. All patients signed the informed consent, and the experiment was approved by the Ethics Committee of The Affiliated Hospital of Hangzhou Normal University.

### The establishment of the sepsis model

All animal experiences were approved by the Laboratory Animal Management Committee of Hangzhou Normal University Laboratory Animal Center.

A total of 12 adult clean Kunming mice aged 6-8 weeks and weighing 18-22g were provided by the Laboratory Animal Center of Chongqing Medical University (Chongqing, China). All mice were raised independently at 20-25°C and 50% humidity, and then were randomly divided into two groups: the sham group and the CLP group. Animals were fasted one day before the experiment and anesthetized with 0.3% sodium pentobarbital (30 mg/kg, Solarbio, Beijing, China). In the midline of the mouse, we cut the mouth about 1cm long, found the cecum and ligatured the root, retained the blood supply, and then used the suture to puncture the ligated cecum through the serosal surface of the blind end of the intestine opposite to the mesentery. Two holes were made once to extrude a little intestinal content, close the abdominal cavity layer by layer with 1-0 silk thread, and immediately subcutaneously inject 1 mL of normal saline to supplement the intraoperative fluid loss^18^ Animals in the sham group were treated in the same manner, but the cecum was not perforated and not ligated.

### Cell culture and transfection

TCMK-1 mouse kidney epithelial cell line was purchased from BioVector NTCC Inc. (Beijing, China) and cultured in 90% high-glucose Dulbecco’s modified Eagle medium (DMEM, Solarbio, Beijing, China) and 10% fetal bovine serum (FBS, Solarbio, Beijing, China) at 37°C. After incubation for 24 h, cells were divided into two groups: one group was added with 5 mg/L of lipopolysaccharide (LPS, Solarbio, Beijing, China) to mimic inflammation, and the other group was added with equal amount of DMEM for 2 h at 37°C as control group.

HEK293 cell line was purchased from American Type Culture Collection (ATCC; Manassas, USA) and cultured in Eagle’s Minimum Essential Medium (EMEM, Solarbio, Beijing, China) with 10% FBS at 37°C for dual luciferase reporter assay.

THBS2-small interfering RNA (siTHBS2), miR-106a inhibitor, miR-106a mimics or their negative control (NC), siNC, NC inhibitor and NC mimics were added to cells cultured in 96-well plate to transfect for 48h at 37°C by Lipofectamine 3000 (Thermo Fisher Scientific, Waltham, USA).

### Cell viability

Cell viability was detected by Cell Counting Kit-8 (CCK-8, MedChemExpress, New Jersey, USA). Briefly, cells (100 μL/well) were seeded in 96-well plate for 24 h, added to 10 μL of CCK-8 solution and incubated in an incubator at 37°C for 3h. The absorbance at 450 nm (OD450) was detected by a microplate reader (Bio-Rad, Hercules, USA).

### Enzyme linked immunosorbent assay (ELISA)

Cultured cells were centrifuged for 10 min to obtain the culture supernatant. The contents of tumor necrosis factor (TNF-α), interleukin 1 beta (IL-1β) and interleukin 6 (IL-6) were detected by ELISA Kit (Abcam, Cambridge, USA) following the instructions. The absorbance at 450 nm (OD450) was measured by a microplate reader.

### Quantitative real-time polymerase chain reaction (qRT-PCR)

Cells were lysed with Biozol Reagent (Biobee, Beijing, China), extracted with chloroform and isopropanol (Sustgreen, Nanjing, China), precipitated in 75% ethanol and washed to obtain total RNA. The synthesis of cDNA was performed using SYBR primeScript^TM^ RT-PCR Kit (TaKaRa, Dalian, China) according to the Kit instructions. The primers were designed by Primer 5.0 software (PREMIER Biosoft International, California, USA) and synthesized by TaKaRa Biotechnology Co., Ltd (Dalian, China). The specific sequences of primers were shown in Table 1. qRT-PCR was performed by SYBR Green I dye method on the PCR instrument according to the operation instructions of ExScript^TM^ RT-PCR Kit (TaKaRa, Dalian, China). Reaction conditions of PCR were as follow: pre-denaturation 95°C for 10s, denaturation 95°C for 5s, fluorescence detection 72°C for 10s, in total of 35 cycles. The data were calculated by 2^-^ method 19.

**Table 1:** The sequence of primers.

Gene	F/R	Primer Sequence (5’ to 3’)
miR-106a	F	AAAAGUGCUUACAGUGCAGGUAG
	R	ACCUGCACUGUAAGCACUUUUUU
TNF-α	F	ACCACGCTCTTCAGCCTACTG
	R	ACGGGCATATCTGAGGTATGAG
IL-1β	F	AGCTGACCCTAAACAGATGA
	R	GATCTACACTCTCCAGCTGTAGC
IL-6	F	CCTCCAGGAACCCAGGTATGAA
	R	TCAGGTGCCCCAGCTACATTAT
THBS2	F	CGTGGACAATGACCTTGTTG
	R	GCCATCGTTGTCATCATCAG

F: Forward; R: Reward

### Western blotting

The total protein was obtained by cells lysis with RIPA Lysis Buffer (Haigene, Harbin, China), separated by sodium dodecyl sulfate-polyacrylamide gel electrophoresis (SDS-PAGE), transferred to nitrocellulose (NC) membrane. Then, the membrane was placed in 5% milk for 2 h at room temperature, washed in phosphate buffer saline (PBS). The B-cell lymphoma-2 (Bcl-2, ab32124), Bcl-2 associated X protein (Bax, ab182733), cleaved caspase-3 (ab2302), β-actin (ab179467) and THBS2 (ab84469) antibodies from Abcam were diluted 1,000 times and added into membranes to incubate at 4°C overnight. And then, anti-rabbit IgG antibody (1:2,000; Abcam) was added to incubate for 30 min at room temperature. Finally, the proteins on the membrane were detected by an imaging system (Bio-Rad, Hercules, USA).

### Dual luciferase reporter assay

miR-106a mimics, NC-mimics, empty pmiR-GLO-NC, THBS2-wild type (WT) and THBS2-mutant (MUT) were transfected HEK293 cells by Lipofectamine 3000, and relative luciferase activity was tested by Dual-Luciferase^®^ Reporter Assay System Protocol (Promega, Madison, USA).

### Statistical analysis

All data were analyzed by SPSS 19.0 software, and students’ *t*-test was used for comparison between the two groups. The difference was significant when p<0.05, and then the results were represented by mean ± standard error (SE).

## Results

### MiR-106a was up-regulated in serum of sepsis patients and CLP-induced mice models

**Figure 1:**
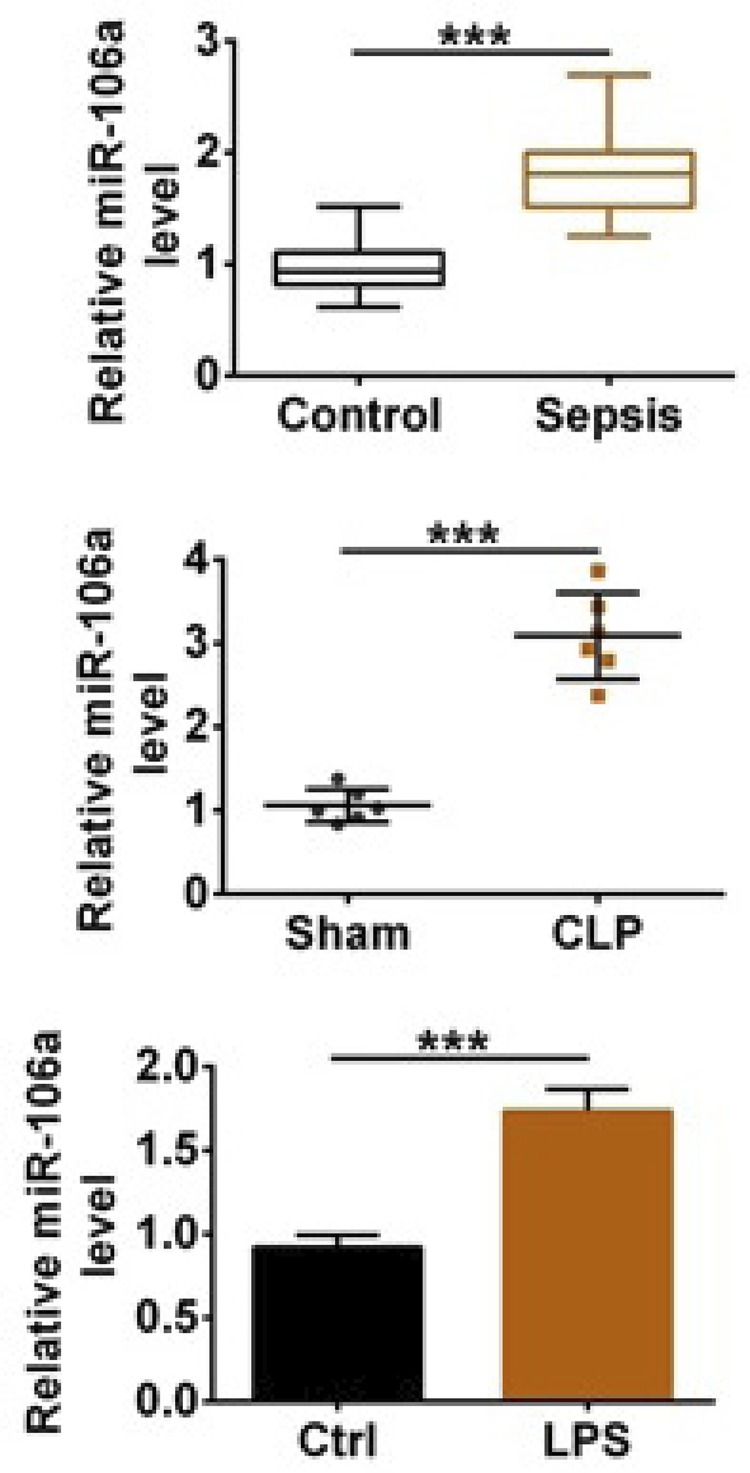
MiR-106a was up-regulated in serum of sepsis patients and CLP-induced mice models. The relative miR-106a level was tested by qRT-PCR in the serum from sepsis and healthy patients, CLP-induced sepsis mouse models and TCMK-1 cells treated with LPS. *** p<0.001.

In order to study the expression level of miR-106a, we collected serum from sepsis patients and healthy patients, established CLP-induced sepsis model in mice and treated TCMK-1 cells with LPS. The results in Figure 1 showed that miR-106a expression level in the sepsis, CLP and LPS group was higher than that in the control or sham group (p<0.001), illustrating that sepsis and sepsis-induced inflammation promoted miR-106a up-regulation *in vivo* and *in vitro*.

### Knockdown of miR-106a reduced LPS-induced apoptosis in TCMK-1 cells

**Figure 2:**
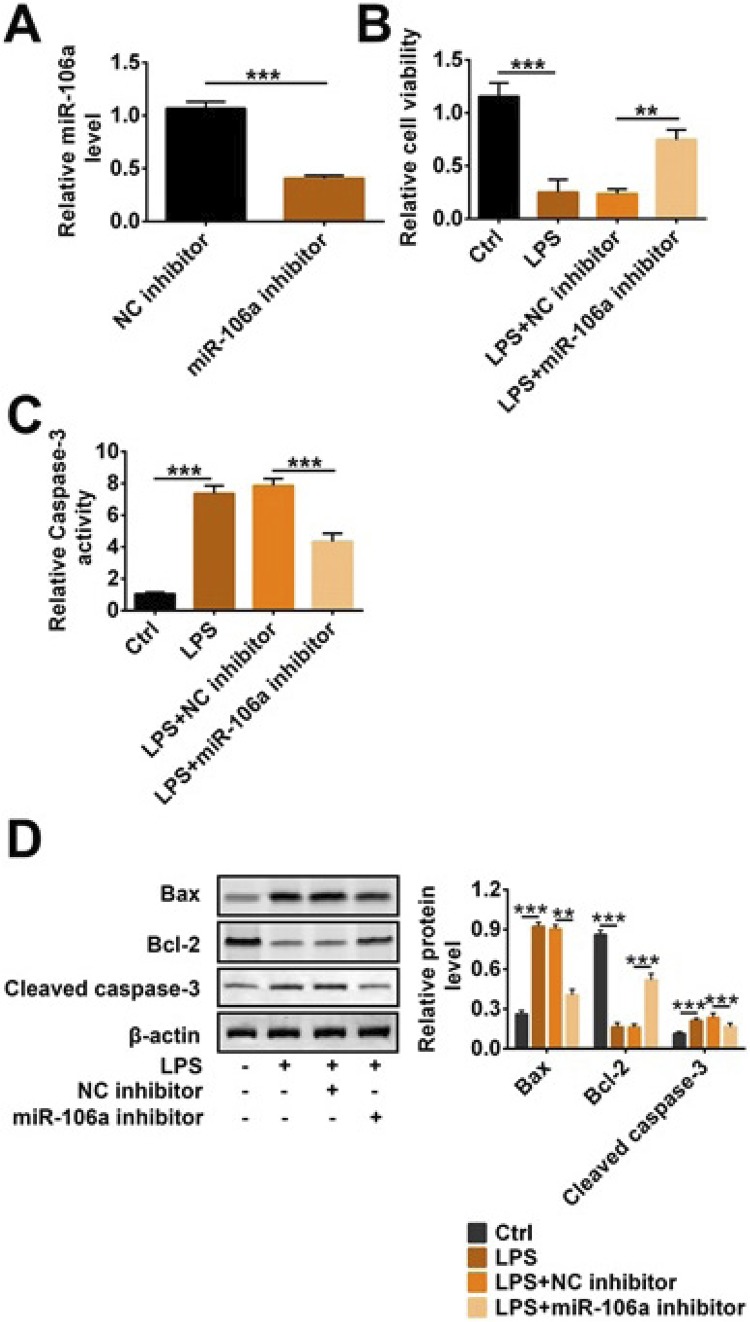
Knockdown of miR-106a reduced LPS-induced apoptosis in TCMK-1 cells. miR-106a inhibitor, NC inhibitor and LPS were transfected into TCMK-1 cells. (A) Relative miR-106a level, (B) cell viability, (C) relative caspase-3 activity and (D) the protein level of Bax, cleaved caspase-3 and Bcl-2 were detected by qRT-PCR, CCK-8 and western blotting. ** p<0.01; *** p<0.001.

In order to investigate the mechanism and effect of miR-106a on sepsis-induced AKI, we used miR-106a inhibitor to transfect TCMK-1 cells treated with LPS. In Figure 2A, miR-106a expression level was significantly reduced in the miR-106a inhibitor group compared with the NC inhibitor group, indicating that miR-106a was efficiently transfected into cells (p<0.001). CCK-8 results in Figure 2B showed that cell viability was decreased in cells treated with only LPS, and this decrease was reversed after adding miR-106a (p<0.01 or p<0.001). On the contrary, caspase-3 activity in the LPS group was higher than that in the control group (p<0.001, Fig. 2C). But caspase-3 activity in the LPS+miR-106a inhibitor group was less than that in LPS+NC inhibitor (p<0.001, Fig. 2C). Moreover, western blotting results showed that the expression of Bax and cleaved caspase-3 were clearly increased and Bcl-2 expression was reduced when adding LPS; conversely, miR-106a inhibitor alleviated these increase and reduction (p<0.001, Fig. 2D). Above data indicated that miR-106a knockdown increased cell viability and decreased apoptosis-related protein expression in LPS-induced TCMK-1 cells.

### Knockdown of miR-106a reduced LPS-induced inflammatory factor levels in TCMK-1 cells

**Figure 3:**
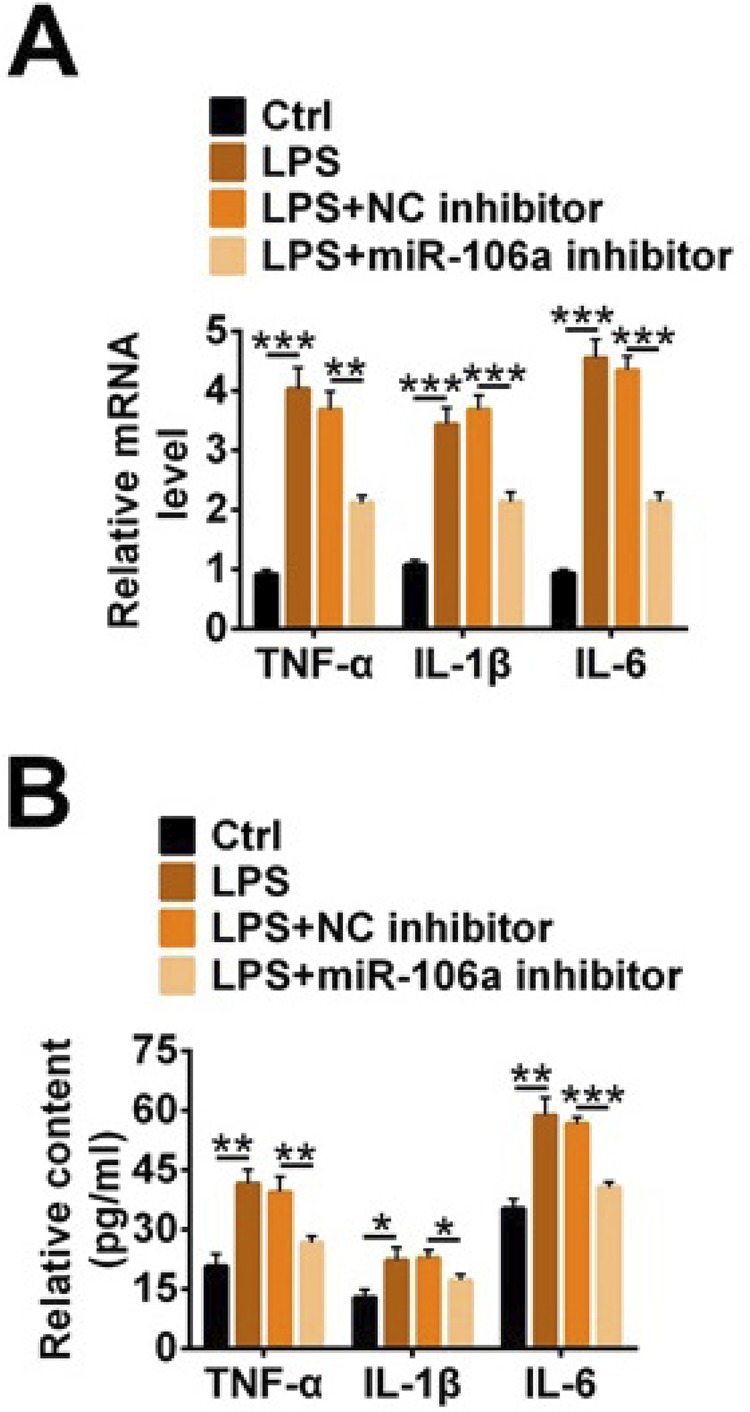
Knockdown of miR-106a reduced LPS-induced inflammatory factor levels in TCMK-1 cells. miR-106a inhibitor, NC inhibitor and LPS were transfected into TCMK-1 cells. (A) The relative mRNA level and (B) content of TNF-α, IL-1β, IL-6 were detected by qRT-PCR and ELISA. * p<0.05; ** p<0.01; *** p<0.001.

In order to further investigate the effects of miR-106a on sepsis, we selected three cellular inflammatory factors TNF-α, IL-1β, IL-6 as representatives for research. The results from qRT-PCR and ELISA in Figure 3A and B displayed that TNF-α, IL-1β, IL-6 expression and content were significantly increased in the LPS group compared with the control group (p<0.05, p<0.01 or p<0.001). After adding miR-106a inhibitor to LPS-induced cells, the expression and content of TNF-α, IL-1β, IL-6 were reduced, suggesting that miR-106a knockdown mitigated the increases of TNF-α, IL-1β, IL-6 caused by LPS.

### THBS2 was a target of miR-106a

**Figure 4:**
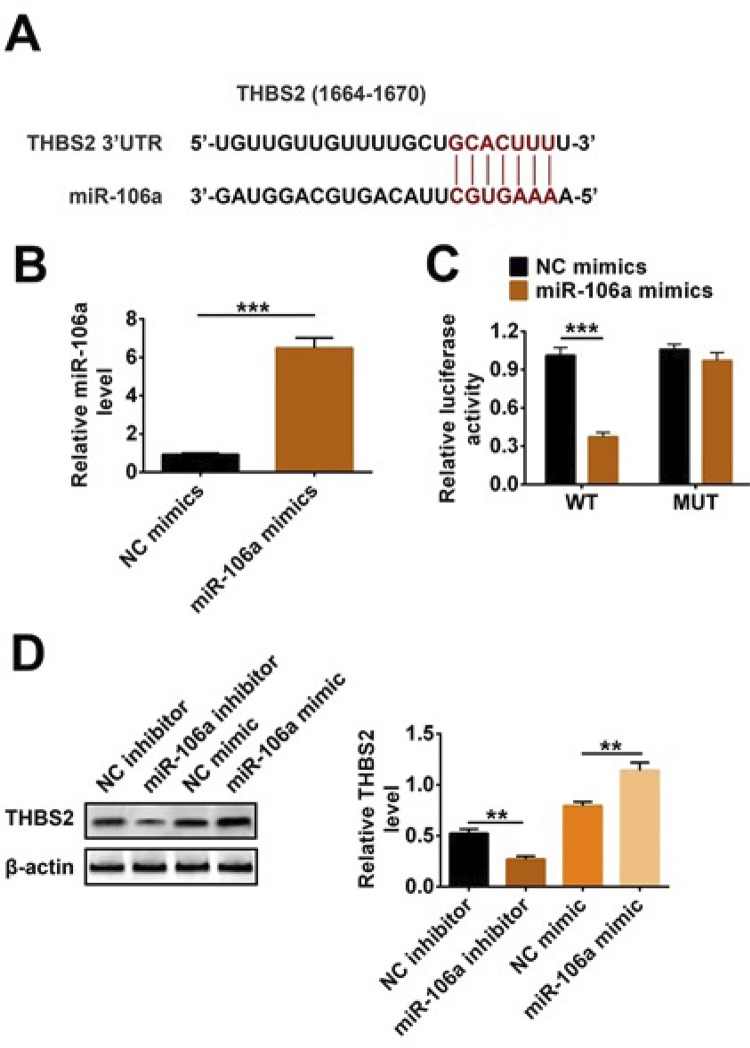
THBS2 was a target of miR-106a. MiR-106a inhibitor, NC inhibitor, and miR-106a mimics, NC mimics, THBS2-WT and THBS2-MUT were transfected into HEK293 and TCMK-1 cells. (A) The binding site was predicted by Targetscan website. (B) Relative miR-106a level was tested by qRT-PCR. (C) The target relationship between miR-106a and THBS2 was confirmed by dual luciferase reporter assay. (D) THBS2 expression was detected by western blotting. ** p<0.01; *** p<0.001.

In this study, we used TargetScan website (http://www.targetscan.org) to find a potential target gene THBS2 downstream of miR-106a, and their binding site was shown in Figure 4A. Then, we also used double luciferase reporter assay to identify the targeting relationship between THBS2 and miR-106a. Figure 4B displayed that miR-106a mimics were successfully transfected into cells (p<0.001). In Figure 4C, relative luciferase activity in the miR-106a mimics+THBS2-WT group was less than that in the NC mimics+THBS2-WT group (p<0.001). However, relative luciferase activity did not change in the miR-106a mimics+THBS2-MUT group. The results in Figure 4C suggested that there was a targeting relationship between THBS2 and miR-106a. Furthermore, western blotting results showed that THBS2 expression level was up-regulated in the miR-106a inhibitor group in relation to the NC inhibitor group; conversely, it was clearly decreased when miR-106a overexpression in TCMK-1 cells (p<0.01). Hence, these results illustrated that THBS2 was a target of miR-106a and negatively regulated by miR-106a.

### MiR-106a increased LPS-induced inflammation and apoptosis of TCMK-1 cells via regulating THBS2 expression

**Figure 5:**
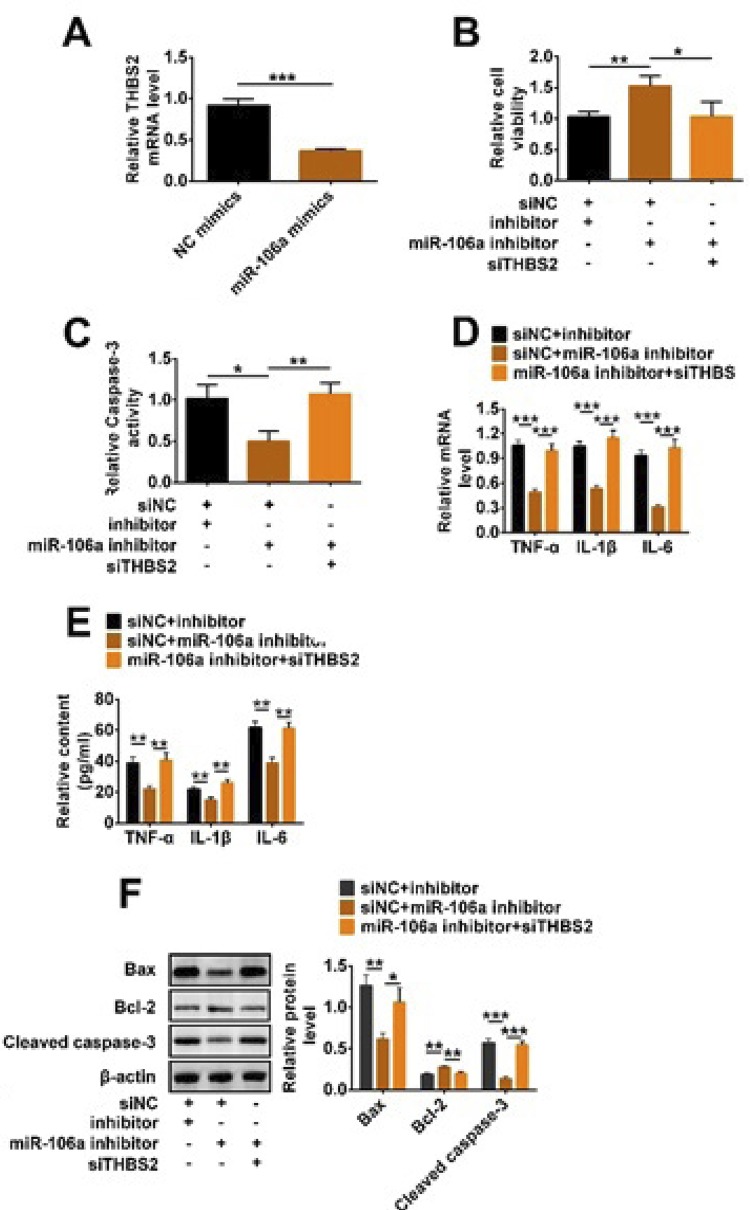
MiR-106a increased LPS-induced inflammation and apoptosis of TCMK-1 cells via regulating THBS2 expression. MiR-106a inhibitor, NC inhibitor, and miR-106a mimics, NC mimics, siTHBS2 and siNC were transfected into LPS-treated TCMK-1 cells. (A) Relative THBS2 mRNA level, (B) cell viability, (C) relative caspase-3 activity, (D-E) the relative mRNA level and content of TNF-α, IL-1β, IL-6, as well as (F) the protein level of Bax, cleaved caspase-3 and Bcl-2 were detected by qRT-PCR, CCK-8, ELISA and western blotting. * p<0.05; ** p<0.01; *** p<0.001.

In order to study how miR-106a and THBS2 together affect sepsis-induced AKI *in vitro*, we used a complement experiment and simultaneously transfected siTHBS2 and miR-106a inhibitor into the LPS-treated TCMK-1 cells. qRT-PCR results in Figure 5A showed that THBS2 mRNA level was reduced in the miR-106a mimics group, indicating that there was a negative regulatory relationship between miR-106 and THBS2. Then, the increase of cell viability caused by miR-106a knockdown was decreased after adding siTHBS2 (p<0.05, Fig. 5B). On the contrary, siTHBS2 alleviated miR-106a knockdown-induced the reduction of caspase-3 activity (p<0.05, Fig. 5C). Furthermore, the mRNA level and content of TNF-α, IL-1β, IL-6 in the miR-106a inhibitor+siTHBS2 group were significantly more than that in the siNC+miR-106a inhibitor (p<0.01 or p<0.001, Fig. 5 D-E). Finally, western blotting results in Figure 5F showed that the down-regulations of Bax and cleaved caspase-3, and the up-regulation of Bcl-2 were reversed by siTHBS2 (p<0.01 or p<0.001). Sum up, miR-106a decreased cell viability and increased apoptosis by regulating THBS2 expression in LPS-induced TCMK-1 cells.

## Discussion

Sepsis is a disordered state in which an inflammatory response is out of control due to severe infection^3^. In recent years, it has been found that miRNAs can regulate the imbalanced inflammatory response by down-regulating inflammatory factors at the post-transcriptional level, suggesting that miRNAs play a very important role in inflammatory response and immune regulation of sepsis^5^. The latest findings confirm that the expression of miRNAs in serum of sepsis patients is also different from that of healthy people^6,7,20,21^. Vasilescu *et al*.^20^ used microarray detection technology to compare the differences in miRNA expression profiles in sepsis patients with normal human serum, and then the results showed that the expressions of miR-150, miR-182 and miR-486 were significantly different in patients with sepsis. Wang *et al*.^21^ reported that miR-146a and miR-233 expressions were reduced in serum of patients with sepsis. Moreover, Wu *et al*.^9^ displayed that the expression of miR-106a was up-regulated in the serum of sepsis mice and inflammatory bowel disease mice model. However, our results in this study were consistent with Wu’s report, that is, we found that the level of miR-106a was increased in the serum of sepsis patients, CLP-treated mice models and LPS-induced cells. These results suggested that miR-106a might be an ideal indicator for the detection and treatment of sepsis or sepsis-induced AKI. Furthermore, a research showed that the absence of miR-106a could alleviate the inflammatory response^10^. It has also been reported that miR-106a can inhibit cell proliferation and promote apoptosis in astrocytoma^22^. Hence, we detected LPS-stimulated TCMK-1 cell viability and apoptosis-related proteins expression, and found that miR-106a knockdown decreased apoptosis and increased cell viability, illustrating that miR-106a could involve in the process of sepsis-induced AKI.

During the recurrence and development of sepsis, the release of cytokines shows cascade effect, including the pro-inflammatory factors TNF-α, IL-1β and IL-6^23,24^. Then, low levels of TNF-α, IL-1β, IL-8 and IL-6 could enhance cellular immune responses, activate defensive function and reduce the incidence of infection^24-26^.

TNF-α has the characteristics of early rapid synthesis and release, and its content changes can reflect the changes of other inflammatory factors^23^. In addition, IL-6 in serum is a delayed cytokine, which can be used as a marker of cytokine cascade activation, reflecting the relationship between host inflammatory response and severity of disease, and also can be used as a prognostic indicator in sepsis^27^. In this study, the results showed that knockdown of miRNA-106a reduced LPS-induced the levels of inflammatory factor TNF-α, IL-1β and IL-6, suggesting that inhibiting the expression of miRNA-106a might inhibit the occurrence of AKI induced by sepsis.

THBS2 is considered to be a powerful angiogenesis inhibitor and a regulator of tissue remodeling after trauma. In recent years, studies have shown that THBS2 plays an important role in tumors, but no studies related to sepsis have been reported^14-16^. However, Wei *et al*.^14^ found that miR-221-3p could increase metastasis of cervical cancer by targeting THBS2. Then, we used TargetScan website to predict the potential target gene of miR-106a and found that there were seven binding sites between miR-106a and THBS2. Furthermore, we also confirmed THBS2 was a target gene of miR-106a and negatively regulated by miR-106a. In addition, our results showed that miR-106a increased LPS-induced inflammation and apoptosis of TCMK-1 cells via regulating THBS2 expression.

In conclusion, miR-106a aggravates sepsis-induced AKI by targeting THBS2 in mice model and cells. MiR-106a might be an ideal test and evaluation index, and further research on the downstream targeting gene THBS2 might provide a broader approach and method for the diagnosis and treatment of AKI caused by sepsis, the development of molecular targeted drugs and prognosis of sepsis patients.

## Footnotes

1 Research performed at The Affiliated Hospital of Hangzhou Normal University, Hangzhou, Zhejiang Province, 310015, China.
